# Fluorescein Application in Cranial and Spinal Tumors Enhancing at Preoperative MRI and Operated With a Dedicated Filter on the Surgical Microscope: Preliminary Results in 279 Patients Enrolled in the FLUOCERTUM Prospective Study

**DOI:** 10.3389/fsurg.2019.00049

**Published:** 2019-08-13

**Authors:** Jacopo Falco, Claudio Cavallo, Ignazio G. Vetrano, Camilla de Laurentis, Lampros Siozos, Marco Schiariti, Morgan Broggi, Paolo Ferroli, Francesco Acerbi

**Affiliations:** ^1^Department of Neurosurgery, Fondazione IRCCS Istituto Neurologico Carlo Besta, Milan, Italy; ^2^Department of Neurosurgery, Barrow Neurological Institute, St. Joseph's Hospital and Medical Center, Phoenix, AZ, United States

**Keywords:** biopsy, brain tumors, central nervous system, fluorescein, neuro-oncology, spinal tumors, YELLOW 560

## Abstract

**Objective:** Sodium fluorescein, a green, water soluble dye, is used as neurosurgical fluorescent tracer thanks to its property to accumulate in cerebral regions of blood-brain barrier (BBB) disruption. The authors report the preliminary results of a prospective observational study regarding the use of fluorescein-guided technique for the resection of suspected malignant neoplasms of the central nervous system (CNS), contrast enhancing at preoperative magnetic resonance imaging (MRI), using a dedicated filter on the surgical microscope.

**Methods:** In March 2016 the authors started a prospective, observational trial to evaluate intraoperative fluorescence's characteristics of CNS tumors, the percentage of extent of resection thanks to fluorescein aid and side effects related to fluorescein administration. This report is based on a preliminary analysis of the results of first 279 enrolled patients. Fluorescein was intravenously injected after intubation or immediately at the entrance in the operating room for awake procedures; the tumor was removed using a dedicated filter on the surgical microscope in an inside-out fashion until all fluorescent tissue was removed, as considered feasible by the surgeon.

**Results:** The 279 patients finally enrolled in the trial, both firstly diagnosed and recurrent, were categorized according to WHO pathological classification and there were 212 neuroepithelial tumors, 25 brain metastases, 10 cerebral lymphomas, 7 hemangioblastomas, or hemangioendotheliomas and 25 other tumors and conditions. No adverse reaction related to the administration of fluorescein or to the combined use of fluorescein with other fluorophores was registered. Fluorescein accumulated in cerebral regions where the BBB was damaged, representing a significant surgical aid in most of the CNS tumors with contrast enhancement. In cases of complete removal of all fluorescent tissue, as intraoperatively judged by the surgeon, postoperative MRI revealed a gross total resection in 181/198 patients (91.4%).

**Conclusions:** Based on these preliminary results, fluorescein-guided surgery with a dedicated filter on the microscope is a safe and effective technique to improve visualization and resection of different CNS tumors and conditions, based on BBB alteration.

## Introduction

Even if Central Nervous System (CNS) tumors do not represent one of the most frequent kind of cancers, they are a major cause of cancer-related mortality and morbidity (1), with an annual incidence in the Western countries of 16.5 per 100.000 adults and of 2.4 new cases per 100.000 children per year. Surgery is the most important treatment in almost all aggressive brain tumors (2–4). The extent of resection (EOR) is a fundamental topic in neurosurgery; if feasible and safely achievable, the gross total resection (GTR) is the most important predictor of overall survival both in high-grade gliomas (HGG) and in metastases (2, 3, 5–9). Furthermore, GTR reduces the risk of recurrence of low-grade gliomas (LGG) or other benign tumors (10, 11). For solitary metastasis, surgical resection plus adjuvant radiotherapy, in form of whole brain radiotherapy or stereotactic radiosurgery, is considered the gold therapeutic standard (12–14); complete metastases resection associated to a favorable factor for prolonged survival. Also for other astrocytomas or rarer primary brain tumors, such as medulloblastomas or ependymomas, radical resection has been correlated to a better prognosis: regarding pilocytic astrocytoma (PA) and ependymomas, a complete resection can achieve local control improving the survival (15, 16).

This need for a radical tumor removal allowed the flourishing of many tools to better visualize the tumor tissue, improving the extent of resection, such as neuronavigation, intraoperative magnetic resonance imaging (MRI), and intraoperative ultrasounds with contrast-enhanced ultrasound (17–19). Among these, fluorophores have acquired an important role thanks to their ability to discriminate between pathological and normal tissue (20–22). Fluorescein is a green fluorescent synthetic organic compound which has countless medical applications (23–27). In form of fluorescein sodium salt (SF) it represents a water-soluble dye with a major blue excitation peak in the region of 460–500 nm and a major green emission peak in the region of 540 to 690 nm.

Although initially its use has been limited to ophthalmology, with historical and sporadic utilization in neurosurgery (28–30), since the introduction of a dedicated and integrated filter in the surgical microscope (YELLOW 560 –*Pentero 900* and *Kinevo; Carl Zeiss Meditec, Oberkochen, Germany*), SF applications in neurosurgery have increased exponentially (31–42). SF, differently to 5-aminolevulenic acid (5-ALA) which is a metabolic tracer (20), could be considered as a vascular fluorophore and its use as fluorescent tracer in neuro-oncological procedures relies on the property to accumulate in pathological areas of blood-brain barrier (BBB) disruption, as in gliomas, but also in other brain tumors, similar to the process of contrast enhancement (c.e.) on MRI.

In July 2015, based upon preliminary scientific results from different studies published in the international literature (34, 36, 37), including a prospective phase II trial from our group (32, 33, 38, 43–46) the Italian Medicine Agency (AIFA) has extended the indications for the utilization of fluorescein molecule (determination 905/2015, Gazette n.168, 22 July, 2015; http://www.gazzettaufficiale.it/atto/serie_generale/caricaDettaglioAtto/originario;jsessionid=izVcTOmnjOzfNRjjw56kAA__.ntc-as2-guri2b?atto.dataPubblicazioneGazzetta=2015-07-22&atto.codiceRedazionale=15A05620&elenco30giorni=false). According to this determination, the intravenous (i.v.) injection of SF as a neurosurgical tracer during oncological procedures for aggressive tumors of the CNS is approved and its cost are totally reimbursed by the Italian National Health System. Therefore, in March 2016, the authors started a new prospective observational study, called FLUOCERTUM (FLUOrescein in CERebral TUMors), regarding the use of SF as a fluorescent intra-operative tracer in patients with suspected aggressive tumors of the CNS. In this paper, we present the preliminary results of the first 279 patients enrolled in this study, until December 2018.

## Methods

### Patients and Inclusion Criteria

The inclusion criteria were as follows: (1) patients of both genders, at any age; (2) patients with suspected aggressive lesions of the CNS, as suggested by preoperative MRI or computerized tomography (CT) with i.v. contrast agent administration. The exclusion criteria were: (1) impossibility to give consent due to cognitive deficits or language disorders; (2) known allergy to contrast agents or history of previous anaphylactic shocks; (3) known severe previous adverse reactions to SF; (4) acute myocardial infarction or stroke in the last 90 days; (5) severe renal failure; (6) severe hepatic failure; (7) severe heart failure; (8) women in their first trimester of pregnancy or lactation.

The study started in March 2016, when the first patient was enrolled. Written informed consent was obtained from all patients. The FLUOCERTUM study has been approved by the Ethical Committee of the Fondazione IRCCS Istituto Neurologico Carlo Besta.

Three hundred and twenty-eight patients were screened for participation in this prospective trial. Two patients were excluded from the analysis because they refused surgery, seventeen patients were excluded because SF was not intraoperatively injected, while fourteen patients were excluded because the specific YELLOW 560 filter of the microscope was not available or not working properly; finally, we excluded sixteen patients because surgery was performed under 5-ALA guidance but SF was administered only for confocal laser endomicroscopy evaluation. The final enrolment comprised 279 patients: 252 adult patients (145 male and 107 female) from 18 to 82 years (mean age 52.1 years) and 27 pediatric patients (14 male and 13 female) from 2 to 17 years (mean age 9.82 years).

### Clinical and Radiological Management

As part of normal clinical practice at our Institution, preoperative assessment included physical and neurological examination, laboratory tests results, preoperative contrast-enhanced MRI or CT scans for neuronavigation, recording of concomitant medications (as steroids) and previous radiation therapies. In pre-operative MRI, patients were categorized based on pre-operative contrast enhancement characteristics and divided in cases with clear contrast enhancement and cases with only minimal/absent contrast enhancement. To evaluate the EOR, a volumetric MRI examination was performed for each patient within 72 h after surgery; in particular, to calculate the residual pathological volume, the hyperintense alterations in volumetric basal T1 acquisitions were subtracted from the volume of hyperintense tissue in post-contrast volumetric T1 images, to avoid the incidental inclusion of blood or blood product. The post-operative clinical evaluation included a standard neurological examination and exclusion of occurrence of any side effect related to fluorescein injection. In particular, according to AIFA determination, arterial blood pressure, heart rate, blood oxygen saturation, temperature, and skin color were monitored pre-operatively and three times per day during the first three post-operative days; plasmatic dosage of creatinine was controlled the first and third post-operative day. The follow-up considered in this trial ended at the completion of the immediate post-operative radiological examination and clinical observation, as in AIFA determination.

### Surgical Protocol

Our surgical protocol of fluorescein-guided technique is the same used in the FLUOGLIO trial since 2012 and it has already been described in previous papers (32, 33, 38), and based on i.v. SF (*Monico SpA, Italy*) injection at standard dose of 5 mg/kg, by a central venous line, immediately upon completion of the induction of general anesthesia or anesthetic procedures in awake surgery. The surgery was performed with the aid of a surgical microscope equipped with an integrated fluorescent filter specific for fluorescein (*Pentero* or *Kinevo* microscopes, with YELLOW 560 filter; *Carl Zeiss Meditec, Oberkochen, Germany*). During resection, the microscope could be switched alternatively from fluorescent to white-light illumination; neuronavigation, intraoperative ultrasounds, and contrast-enhanced ultrasound or other tools could be used according to the surgeon's preference. In tumors located in eloquent areas, intraoperative neurophysiological monitoring was used. Tumors were removed in an inside-out fashion until all fluorescent tissue was removed, as considered feasible by the surgeon.

In cases where tumor vessels, or peritumoral arteries and veins needed to be intraoperatively evaluated, indocyanine green (ICG) video-angiography with FLOW 800 analysis (*Carl Zeiss Meditec, Oberkochen, Germany*) and, sometimes, ICG temporary clipping test were performed, as already reported (47–51).

### Histological Analysis

Histopathological analysis was performed in each case; tumors were classified according to the 2016 WHO classification by the neuro-pathology group of our Institute, with no additional costs respect to clinical practice (52).

### Statistical Analysis

The study population was classified about gender, age, histological subtype, tumor volume and location, and therefore evaluated with descriptive analysis. Fluorescence intensity was graded by the surgeon as intense/bright, inhomogeneous or homogeneous, moderate or slight/absent; surgeons were also asked to classify the use of SF per each procedure as helpful/useful, not essential, not helpful/useless to achieve surgical aims. The EOR was prospectively calculated as a percentage of tumor resection based on early contrast-enhanced postoperative MRI or CT imaging; according to the entity of resection, we distinguished five main categories: GTR (EOR 100%), sub-total resection (STR, with an EOR of 90–100%), partial resection (PR, 30–90%), open biopsy and frameless/stereotactic biopsy. Rate of removal was studied in the different histological subtypes and compared with ANOVA by Prism software.

## Results

All the 279 patients finally enrolled in the trial were classified and categorized as stated by the 2016 WHO central nervous system tumors classification; the results about intraoperative fluorescence characteristics, surgical utility and extent of resection, were then related to the different histotypes.

Briefly, there were 212 neuroepithelial tumors, 25 brain metastases, 10 cerebral lymphomas, 7 hemangioblastomas or hemangioendotheliomas and 25 other tumors and conditions in differential diagnosis or in which SF represented a surgical aid (Tables 1, 2).

**Table 1:** Intraoperative fluorescence characteristics and utility, based on tumor histology, in the surgical group.

**HISTOLOGIES**		**Number of patients**	**Intraoperative fluorescence**	**Extent of resection**				**Surgeon's opinion**
				**GTR (# - %)**	**Unexpected STR (no fluorescent residual tissue–not enhancing tumor)(# - %)**	**Expected STR (fluorescent residual tissue) (# - %)**	**PR (# - %)**	
Tumors of neuroepithelial tissue	Diffuse astrocytic and oligodendro	143	- Heterogeneously intense (HGG) - Absent, only some spots (LGG)	104 (72.7)	13 (9.1)	19 (13.3)	7 (4.9)	- Helpful in HGG (132 cases) - Useless in LGG (11 cases)
	Ependymoma	14	Homogeneously intense	10 (71.5)	1 (7.1)	2 (14.3)	1 (7.1)	Helpful (14 cases)
	Glioneuronal	13	- Homogeneously intense - Slight/Absent (rosette-forming glioneuronal tumor)	9 (69.2)	1 (7.7)	3 (23.1)	0	- Helpful (9 cases) - Partial utility during surgery for drug-resistant epilepsy (3 cases) - Useless in rosette-forming glioneuronal tumor (1 case)
	PA and other astrocytic	18	- Heterogeneously intense (bright cystic fluid if present) - Homogeneously intense (SEGA)	10 (55.6)	2 (11.1)	5 (27.8)	1 (5.5)	Helpful (18 cases)
	Others (Embryonal, PPTID, …)	8	Intense: - Heterogeneously (medulloblastoma, PPTID and angiocentric glioma) - Homogenously (choroid plexus papilloma)	7 (87.5)	0	0	1 (12.5)	- Helpful (7 cases) - Not essential in choroid plexus papilloma (1 case)
Cerebral metastases		25	Heterogeneously intense	24 (96)	0	1 (4)	0	- Helpful (20 cases) - Not essential in 3 skin melanoma metastases and in one hemorrhagic metastasis (4 cases) - Useless in 1 case (previous whole brain radiotherapy and radiosurgery)
Primary CNS lymphomas		4	Homogeneously intense	4 (100)	0	0	0	Helpful (4 cases)
Meningiomas		3	Homogeneously intense	3 (100)	0	0	0	Not essential (3 cases)
Cerebral hemangioblastomas and hemangioendothelioma		7	Moderate (Bright cystic fluid)	7 (100)	0	0	0	Not essential (7 cases)
Tumors of cranial and paraspinal nerves		7	Homogeneously intense	2 (28.6)	0	5 (71.4)	0	Not essential (7 cases)
Tumors of the sellar region		1	Homogeneously intense (motor oil liquid, not enhancing)	1 (100)	0	0	0	Helpful (1 case)
Tumors of soft tissue and bone (Nothocordal)		2	Heterogeneously fluorescent	0	0	2 (100)	0	Not essential (2 cases)

**Table 2:** Intraoperative fluorescence characteristics and utility, based on histopathological results, in the biopsy group.

**Histologies**		**Open biopsy**			**Needle biopsy**		
		**Number of procedures**	**Intraoperative evaluation**	**Diagnostic accuracy**	**Number of procedures**	**Intraoperative evaluation**	**Diagnostic accuracy**
Tumors of neuroepithelial tissue	Diffuse astrocytic and oligodendro	4	Helpful to highlight the target of biopsy in multifocal presentation	100% (GBM)	10	Bright samples visualized under Y560 filter	70% (6 GBM, 1 AA) 1 choroid plexus 2 reactive gliosis
	Pilocytic astrocytoma	1	Helpful to highlight the fluorescent component in a diffuse spinal lesion	100% (PA)	0	/	/
	Pineal region	1	Helpful to obtain the specimen of the cystic wall	100% (Pineocytoma)	0	/	/
Primary CNS lymphomas		5	Helpful to highlight the target of biopsy in multifocal presentation	100% (B-cell NHL)	1	Bright samples visualized under Y560 filter	100% (B-cell Lymphoma)
Vasculitis processes		5	Helpful to highlight the target of biopsy in multifocal presentation	100% (4 Vasculitis and 1 Cerebral Amyloid Angiopathy)	1	Moderate fluorescence under Y560 filter	100% (Vasculitis)

### Tumors of Neuroepithelial Tissue

#### Diffuse Astrocytic and Oligodendroglial Tumors

One-hundred fifty-seven patients underwent surgery, either for removal and for open or stereotactic biopsy. In 142 cases, the tumor presented inhomogeneous contrast-enhancement, with cystic-necrotic areas and solid components; only 15 selected cases of patients with minimal/absent contrast enhancement were scheduled for fluorescein-guided surgery in this subgroup of patients.

In the 142 cases presenting with intense and inhomogeneous contrast-enhancement, 128 underwent surgery for tumor removal and 14 underwent open (4 cases) or needle biopsy 10 (cases).

**Figure 1:**
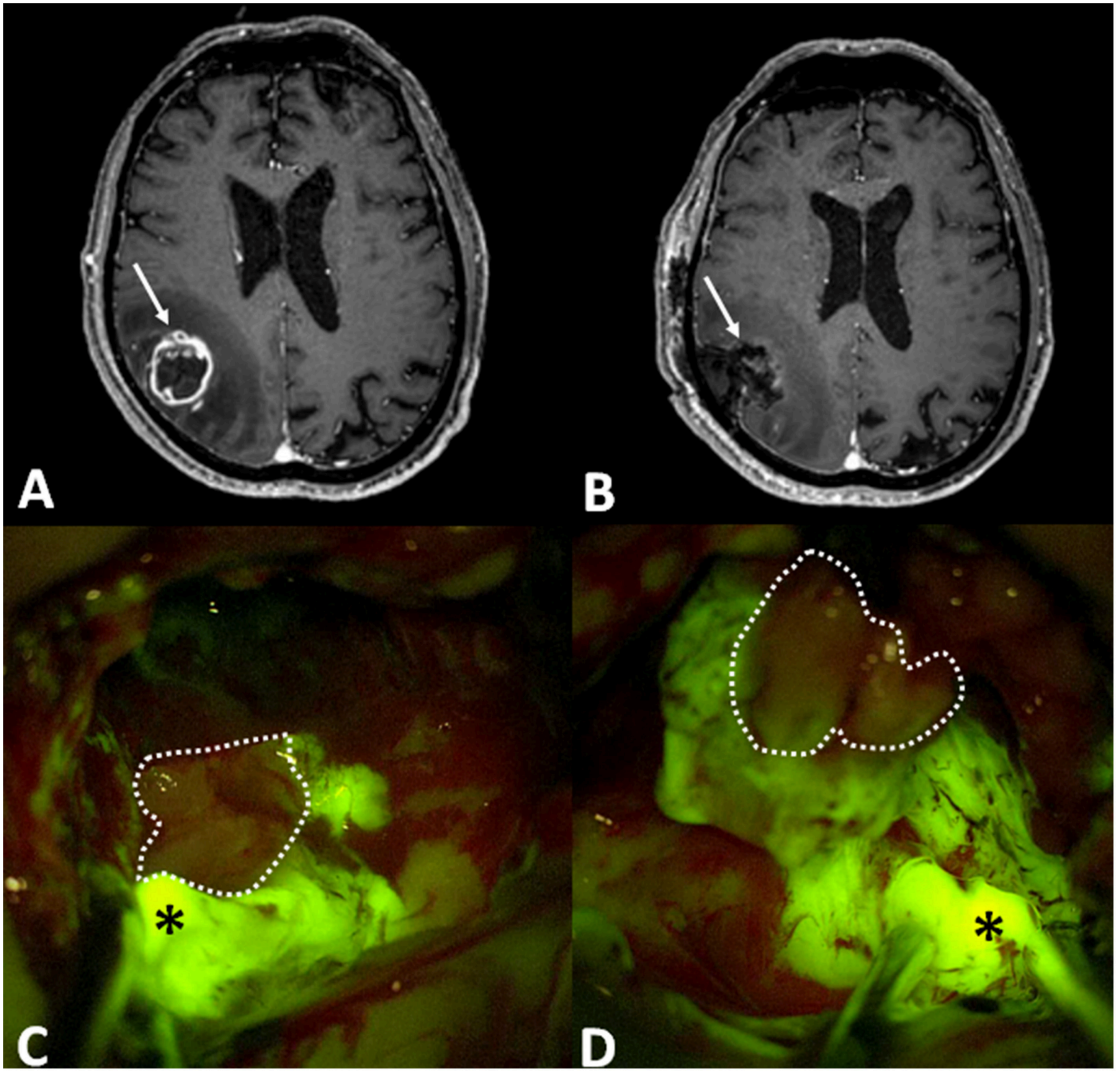
**(A)** Preoperative post-contrast axial T1-weighted MRI scan showing a right parietal GBM (arrow), that was completely removed, as visible in post-operative T1 with contrast axial scan (**B**: in that case arrow is the surgical cavity). Intraoperative images, under Y560 filter **(C,D)**, depict bright yellow green signal (asterisk), in correspondence of nodular component, and dark pinkish feature in the necrotic core (dotted line).

In the 128 cases submitted to tumor removal, the histological diagnosis was HGG in all cases. There were 78 primary and 35 recurrent glioblastoma (GBM), 4 newly diagnosed and 1 recurrent anaplastic astrocytoma (AA), 3 newly diagnosed and 5 recurrent anaplastic oligodendrogliomas (AO), 1 newly diagnosed and 1 recurrent NOS high-grade glioma. Intraoperatively (Figure 1), these tumors usually presented as brightly fluorescent, with a yellow-green signal clearly distinguishable by pinkish-appearing peritumoral parenchyma under Y560 filter (Figures 1C,D). Sometimes, in cases with a clear central necrotic core, this tissue showed a dark pinkish feature with scant fluorescein enhancement (Figures 1C,D). This heterogenous appearance was more pronounced in recurrent tumors, already submitted to radiotherapy, although previous fractionated radiation therapy did not determine substantial difference about fluorescence characteristics or usefulness upon surgical evaluation. Finally, in some cases a bright fluid component was reported which was the result of tumor central colliquation. In all these 128 cases, SF was considered helpful by the operating surgeon to help in distinguishing tumor from healthy tissue (100% of the cases). In 95 out of 128 cases (74.2%), fluorescent tissue was completely removed, and the post-operative MRI confirmed a gross-total resection. In 18 out of 128 cases (14.1%) residual fluorescent tissue was deliberately left at the end of surgery, due to the proximity to eloquent areas or the adherence to major brain vessels; in these cases, an expected subtotal resection was achieved at post-operative MRI. In 11 out of 128 cases (8.6%) fluorescent tissue was intraoperatively judged as completely removed, while postoperative MRI showed an unexpected subtotal resection. In 4 out of 128 cases (3.1%) a partial resection was achieved (Table 1).

SF was used also in 4 cases of multifocal lesions, to guide open biopsy to highlight the area of fluorescent tissue in the context of the tumor. In all of these cases (100%) SF was considered useful and the histological analysis was possible, with a diagnosis of GBM. Finally, in the 10 cases of needle biopsies, the samples were evaluated under the YELLOW 560 filter of the microscope and resulted to be fluorescent in all cases; however, diagnostic accuracy was confirmed in 7/10 (70%) with a histological diagnosis of GBM in 6 cases and AA in 1 case; in three cases histological analysis was not diagnostic (1 choroid plexus and 2 reactive gliosis) (Table 2).

Fifteen patients with minimal/absent contrast enhancement underwent tumor removal by fluorescein-guided technique. In 10 cases MRI showed a very extensive area of T2/FLAIR hyperintense signal abnormality, which suggested an aggressive behavior. In those cases, SF revealed in 6 out of 10 cases only minimal intralesional fluorescent spots. Therefore, SF was generally judged useless because no adjunctive information about residual tumor volume were provided compared to white light illumination. All that cases were histological grade II lesions (3 NOS grade II glioma, 3 diffuse astrocytomas and 4 oligodendrogliomas). Five patients presented with slight contrast enhancement at pre-operative MRI, and a positive 11C-methionine (MET) or fluoro-ethyl-tyrosine (FET) - Positron Emission Tomography (PET). In all cases, SF highlighted the pathological regions with higher metabolism, as suggested by PET findings, and it was judged useful in 4 patients, in which histopathological analysis revealed grade III lesions (AA in 3 cases and AO in 1 case). One patient affected by recurrent oligodendroglioma presented only few fluorescent spots, such as the MET positive regions, and SF was not judged useful during surgical removal (overall 80% of utility in the population of non-Gadolinium enhancing, but PET positive gliomas).

#### Other Neuroepithelial Tumors

Fourteen ependymomas, presenting with homogeneous and moderate enhancement at pre-operative MRI were submitted to fluorescein-guided resection. In all cases, the operating surgeon considered SF as a useful adjunct to better identify tumor-healthy tissues interface. GTR was achieved in 10 cases (71.4%); in one case (7.15%), despite the subjective surgical impression of complete fluorescent tissue removal, postoperative MRI showed tiny neoplastic remnants. In two ependymomas of the fourth ventricle (14.3%), there were small fluorescent residual spots due to extremely strong adherence to surrounding health parenchyma. Furthermore, a partial resection was performed in a very huge, recurrent posterior fossa ependymoma (7.15%), due to the significant bleeding (21.45% of expect subtotal or partial removal).

Thirteen neuronal and mixed neuronal-glial tumors were included in this series. Nine patients presented with enhancement (in 7 cases with a diffuse homogeneous enhancement whereas in the other 2 with a bright nodule associated with enhancing peripheral cyst), while four patients presented with minimal/absent enhancement or with an area of c.e. in the context of a larger not-enhancing tumor at pre-operative MRI. The surgeon found bright and homogeneously fluorescent tissue and SF was judged useful in 12 out of 13 cases, except for one case of a slight enhancing spinal rosette-forming glioneuronal tumor (92.3%). In 6 cases, all fluorescent tissue was removed whereas in 3 patients, small residual tumor was left due to the proximity to eloquent brain areas. Furthermore, in 3 patients scheduled for surgery due to drug-resistant epilepsy affected by supratentorial lesions, located in not-eloquent brain, SF was judged useful to identify the small, pathological nodule but the resection was supramaximal and extended to peritumoral gliosis and dysplastic parenchyma to achieve seizure control. Overall a GTR was achieved in 9 patients out of 12 cases (75%) when SF represented a surgical aid, whereas in 3 cases (25%) an expected STR was achieved (4, 53–56).

**Figure 2:**
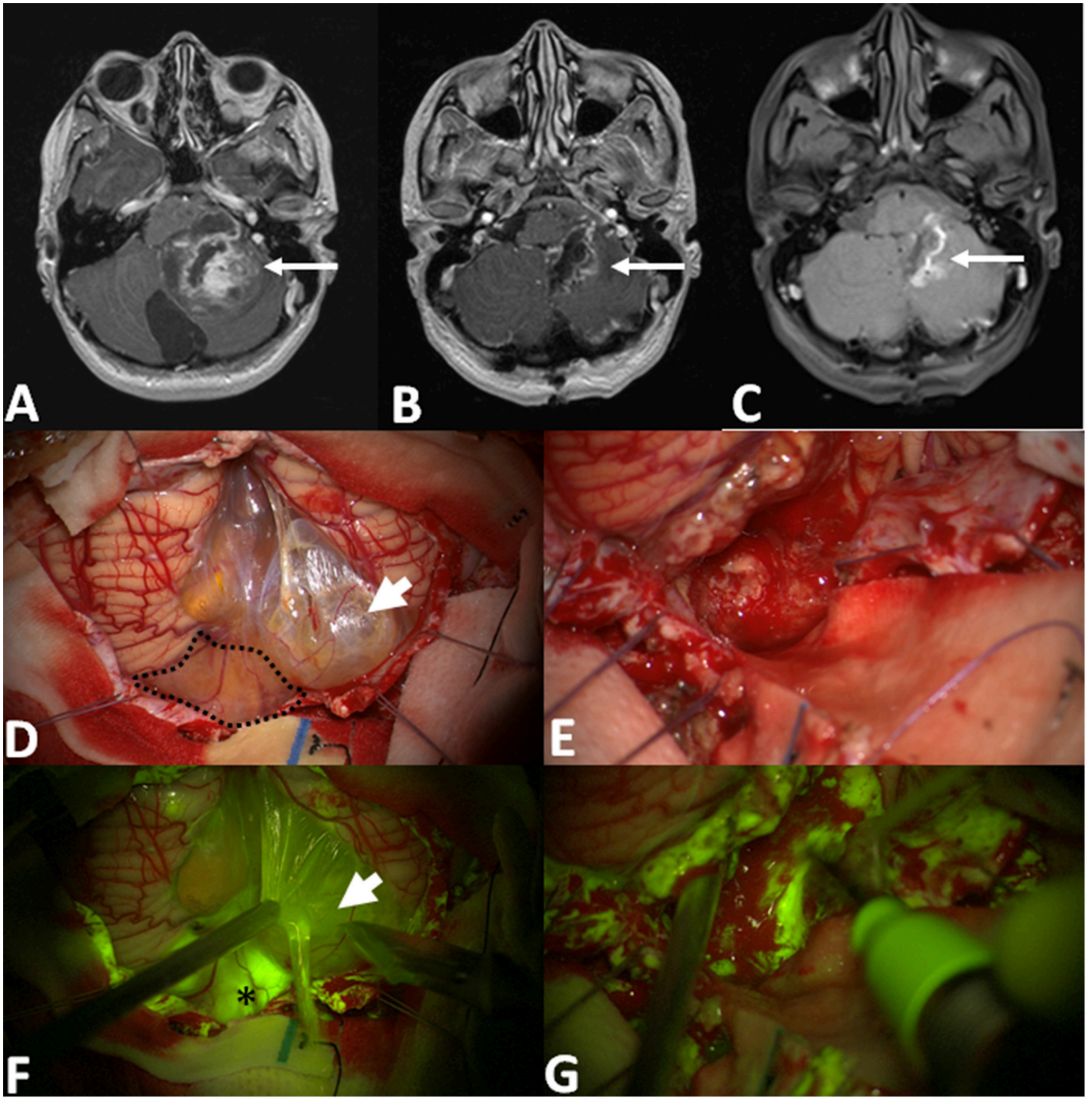
Pre-operative T1 with contrast axial scan **(A)** shows a huge posterior fossa pilocytic astrocytoma (arrow) with a gross-total resection, as detectable by post-operative post-Gadolinium T1 MRI in **(B)**. The area of contrast-enhancement corresponds to the spontaneous hyperintense boundaries of surgical cavity **(C)**. After dural opening **(D,F)**, it is possible to appreciate a multilobulated cyst with a bright fluid (arrowhead in **D**, after its fenestration in **F**) and an inferior vermis nodular component (dotted line in **D**) with an intense fluorescein enhancement (asterisk in **F**). Intraoperative images, under white light **(E)** and Y560 filter **(G)**, during tumor removal with cavitron ultrasound aspirator, showing a bleeding, friable tissue with inhomogeneous fluorescein enhancement.

Pilocytic astrocytoma, pleomorphic xanthoastrocytoma and sub-ependymal giant cell astrocytoma, presented at pre-operative MRI with well-circumscribed margins, typically having both solid (predominant) and cystic components, with inhomogeneous contrast enhancement at pre-operative MRI. The fluorescein enhancement was typically intense in the vast majority of the solid portion of the neoplasia with characteristic bright fluorescent cystic fluid (Figure 2). GTR was achieved in 10 cases out of 19 patients scheduled for tumor removal (52.6%), in 2 cases a minimal residual volume was highlighted by postoperative MRI despite the intraoperative subjective evaluation of complete tumor removal (10.5%); in other 7 cases, the resection was subtotal with fluorescent residual spots to avoid neurological worsening (expected STR in 36.9%). In one case of spinal PA an open biopsy was performed: the most fluorescent component was removed for histological analysis which was diagnostic; the patient did not present any neurological sequelae.

For other neuroepithelial tumors (one choroid plexus papilloma, one recurrent angiocentric glioma and two medulloblastomas), all presenting with inhomogeneous contrast enhancement at pre-operative MRI, intraoperative fluorescein enhancement was intense and judged useful in all cases, even if in the case of choroid plexus papilloma, the tumor tissue was easily recognizable also under white light. SF represented an important surgical aid during pineal surgery, in two intermediate differentiation tumors of the pineal parenchyma, in one papillary pineal tumor and in one cerebellar metastasis of papillary pineal tumor. In these cases, preoperative MRI with Gadolinium showed an intense, inhomogeneous enhancement with cystic regions. Furthermore, SF was used in an open biopsy of a pineocytoma with a voluminous cystic component after its surgical fenestration; radiologically, it appeared as a voluminous cyst with peripheral enhancement. Despite histological differences, all those tumors had a strong and bright fluorescent enhancement and the Y560-guided surgery resulted really useful to identify pathological tissues, preserving normal brain parenchyma.

### Metastatic Tumors

Overall 25 patients with cerebral metastasis had undergone fluorescence-guided surgery since 2016; 13 of them underwent previous radiotherapy/radiosurgical treatment. All lesions presented with typical inhomogeneous contrast enhancement at pre-operative MRI. About the primitive tumors, the majority of them were breast (8), lung (5) and colorectal (3) cancers; 3 seminomas, 3 melanomas and also ovarian, renal and thyroid (one case per each histotypes) were present too. GTR was achieved in 96% of patients (24/25); in the remaining one (4%), a right fronto-mesial metastasis enchasing the ipsilateral callosomarginal artery, the surgeon chose to leave lowercase fluorescent residual tissue around the encased artery.

**Figure 3:**
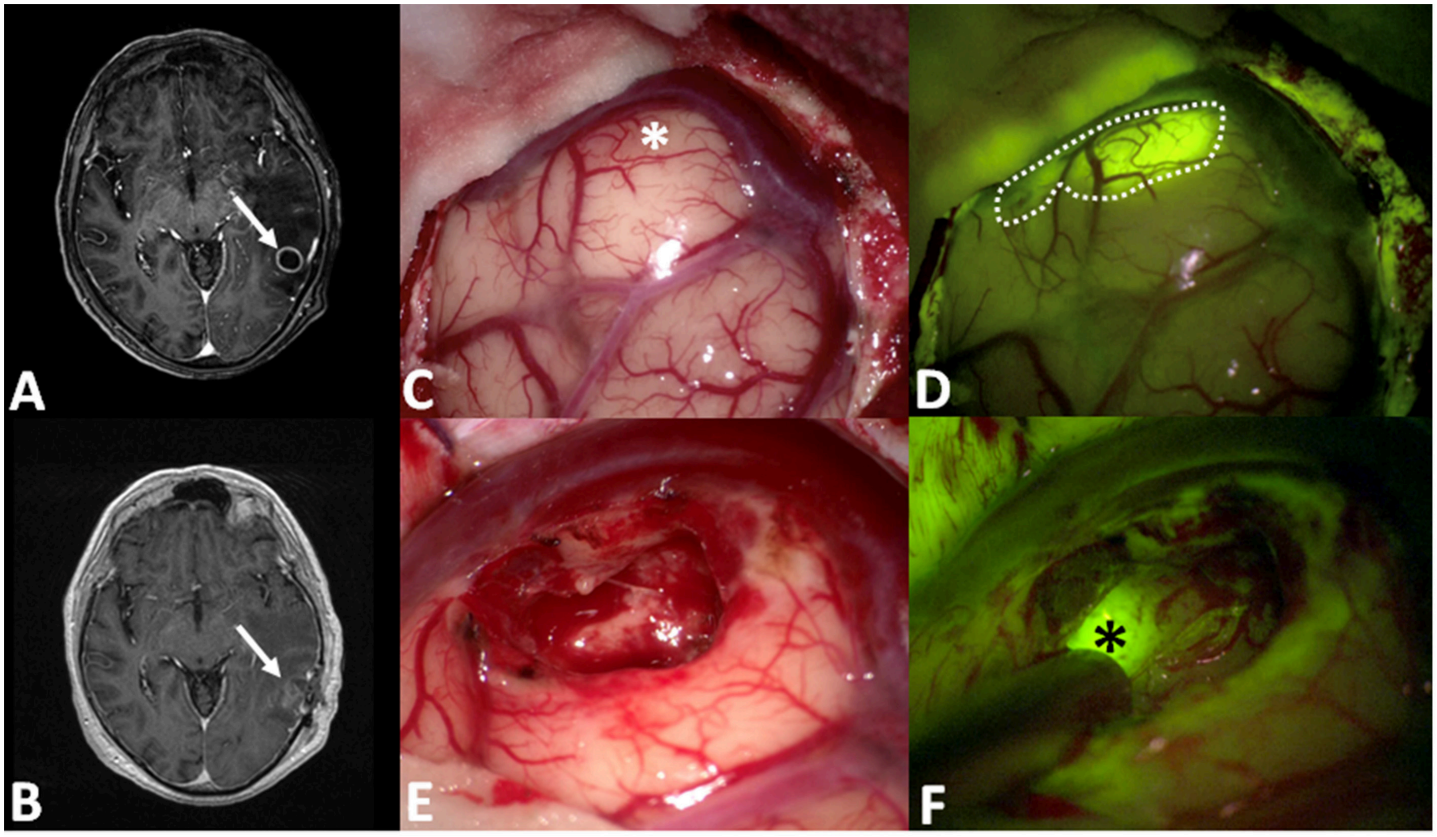
**(A,B)** Pre- and post-operative post-contrast axial T1-weighted scans showing a left temporal metastasis from lung cancer, completely removed. After dural opening **(C)**, the Y560 filter **(D)** can highlight the superficial part of the metastasis, helping the surgeon in discriminating it from peritumoral brain parenchyma (dotted line in **D**), whereas under white light it is possible to appreciate a grayish, vanishing area (asterisk in **C**). During surgical resection **(E,F)**, fluorescein helps the surgeon to identify residual pathological tissue (black asterisks in **F**), not clearly visible under white light **(E)**.

Under YELLOW 560 visualization, metastases appeared intensively fluorescent, with large yellow areas corresponding to the Gadolinium enhancement spots (Figure 3). SF was considered really helpful to distinguish tumor or to identify the neoplastic nodules inside infiltrated parenchyma in 20 procedures (80%). In the 3 patients affected by melanoma (12%), visualization with Y560 filter was not essential in melanoma metastases, that showed a characteristic brown-bluish pigmentation; for the patient with a hemorrhagic metastasis (4%), the filter was activated only at the end of the procedure, to verify the absence of residual fluorescence. Finally, in one patient (4%) with a left frontal colorectal metastasis previously submitted to radiosurgery and whole brain radiotherapy, the lesion appeared strongly fluorescent, but this finding was considered not useful due to fluorescein enhancement of peritumoral parenchyma, secondary to radiation damages.

Furthermore, for 3 superficial metastases, after dural opening and YELLOW 560 activation, the surgeon identified an atypical and greater than usual meningeal fluorescence enhancement, in correspondence of the superficial part of the lesion: in these cases, the dura was sacrificed; the histopathological analysis identified leptomeningeal metastasis.

### Primary Central Nervous System Lymphoma

Ten patients with radiological diagnosis of lymphoma were enrolled: in four of them, surgery was scheduled due to the spinal localization (two cases: one lumbar and one dorsal localization) or huge dimension of a single lesion in non-eloquent brain region (one left posterior temporal and one right parietal). Six patients had a multifocal presentation and were scheduled for open biopsy in five cases and for stereotactic biopsy of the deep, paraventricular, enhancing component in the remaining one; in every case, biopsies were judged diagnostic by the pathologist. Lymphomas, under YELLOW 560 filter visualization, appeared homogenously and brightly fluorescent: fluorescein guidance was always judged useful.

### Cerebral Hemangioblastomas and Hemangioendotheliomas

Six patients with superficial hemangioblastomas and one patient with a left frontal recurrent epithelioid hemangioendothelioma were submitted to fluorescein-guided surgery; GTR was achieved in all cases. Intraoperatively, the nodular component depicted a slight/moderate and homogenous fluorescence; the intra-cystic fluid, however, when present, appeared intensely fluorescent. Despite these findings, fluorescein was considered not essential because that tumor had a peculiar aspect with the typical orange nodule, that made them easily distinguishable from the surrounding structures. We may speculate that, for deep-seated tumors, SF could help to localize the pathological nodule.

### Other Tumors and Conditions

Fluorescein was also used in five vestibular schwannomas, in two lumbar schwannomas and in two chordomas. Although all lesions presented with intense fluorescence, its use for not considered essential to remove the easily recognizable lesion, and because it was not possible to recognize not involved peritumoral cranial or spinal nerve.

SF was used in the only craniopharyngioma approached by craniotomy due to its large dimension and relapse despite the interferon therapy; in this case, the lesion showed an intense and homogeneous fluorescence under the YELLOW 560 filter. SF was considered useful by the surgeon, during the removal, in order to better identify the tumor capsule and to cleave it from the surrounding parenchyma.

SF was also used in three meningiomas: in everyone, it was considered not essential because the tumors were easily recognizable also under white light, even if all showed bright and homogeneous enhancement.

SF was incidentally used in a post-radiation cavernous malformation, located in the superior cerebellar vermis; intra-operatively, after the evacuation of an intra-parenchymal hematoma, the lesion showed an inhomogeneous fluorescence and SF was judged useful to achieve GTR, as confirmed by post-operative MRI, and to distinguish healthy cerebellar tissue.

In other five case, the radiological suspect of cerebral glioma was not confirmed during or after surgery (abscess, inflammatory pseudotumor, radionecrosis with residual pathological vessels of a previously-radiotreated arteriovenous malformation). The abscess appeared heterogeneously, intensely fluorescent; it was intraoperatively recognized under white light, and, after an extemporaneous histopathological evaluation, the procedure was interrupted, and the material sent for bacteriological analysis. Inflammatory pseudotumor was characterized by a diffuse, moderate fluorescein enhancement which was judged useful by the surgeon during lesion removal. Finally, in the case of radionecrosis, the lesion showed a peripherical zone of strong fluorescein enhancement, with a good demarcation from health parenchyma: in this case, SF was judged not essential.

Six patients with a suspected diagnosis of vasculitis or inflammatory angiopathy, presenting with cerebral lesions at least partly enhancing at pre-operative MRI underwent fluorescein-guided open biopsy. In all cases, SF was helpful in recognizing the enhancing lesion under SF, obtaining a fluorescent sample to be analyzed. Fluorescein-guided biopsy was diagnostic in every case: histopathological analysis confirmed the diagnostic suspicion of cerebral vasculitis in five patient and cerebral amyloid angiopathy in the other one.

### Reactions to Fluorescein, to Combined Use of SF With ICG or 5-ALA and Complications

No adverse drug reaction related to SF injection was reported in 309 patients; the only remarkable and visible effect was the transient yellowish staining of urine which disappeared in about 24 h. Moreover, no adverse neurosurgical or clinical event resulted from the combined use of two fluorophores (SF and 5-ALA, SF and ICG) during the same surgical procedure. Preoperative corticosteroid therapy did not affect fluorescence characteristics.

## Discussion

In our surgical series, the utilization of SF represented a useful adjunct in most of the CNS tumors presenting with some degree of contrast enhancement at preoperative neuroimaging scans.

During the last years SF has emerged as intraoperative tracer able to improve brain-tumor visualization, due to its non-specific, vascular mechanism of action related to the accumulation in brain regions with BBB disruption, as it happens with MRI contrast enhancement (34, 36, 38, 57). In fact, independently from their grading, the majority of CNS tumors, except for some grade I and II intra-axial histotypes, determine a BBB damage, thus they have a radiological presentation with contrast enhancement at MRI. Previous experiences had suggested that the use SF could be associated with a bright fluorescence of the tumor area in primary and recurrent HGG, in metastases, in lymphomas, and in spinal intramedullary lesions (34–36, 39). This was also associated with good results in term of extent of resection (39). However, there were in the literature significant variations in SF use, considering dosage and time of injections, in the studies published by different research groups (39).

As the reimbursement of SF as a fluorescent tracer in neuro-oncology has been approved by the Italian Drug Agency (AIFA) in July 2015 (determination 905/2015, Gazette n.168, 22 July, 2015), and based on our extensive experience with HGG (32, 33, 38), we decided to start a prospective observational study on the use of SF for the resection of aggressive tumors of the CNS, applying a standardized protocol, with a dosage of 5 mg/kg and an i.v. injection immediately after patient intubation or at the entrance in the OR for awake craniotomies (58).

As expected, the majority of the cases were HGG (51.6% of the total number of patients included in the study). Most of these patients underwent fluorescein-guided tumor resection based on a high suspect for a high-grade lesion based on pre-operative MRI. In all these cases, SF appeared extremely useful to improve the discrimination between tumor tissue and peritumoral brain parenchyma, with a positive impact in term of EOR, with a percentage of GTR in 74.2%. Coming from an unselected series of patients, these results seems to be extremely interesting, and in line with previous published studies (32, 33, 38, 39, 59).

A particular consideration regards instead the population of patients with gliomas with minimal/almost absent enhancement at preoperative MRI. In all that cases we noted that SF was useless due to the unpredictable, and usually focal fluorescence under the specific Y560 filter, except for the cases of patients with FET/MET-PET positive tumor, as suggested by Schebesch et al. (60). In our series of 5 patients with this characteristic, we could confirm a correlation between the positive PET areas and the fluorescein uptake by tumor tissue in all cases. This finding suggests a predictive ability of PET with amino acid tracers to detect areas with minimal BBB disruption, not shown by pre-operative MRI, which can be intra-operatively identified by using fluorescein-guided surgery. In that cases, SF enhancement could be related to the molecular property of SF which has a slightly lower molecular weight than Gadolinium. The intraoperative recognition of these positive PET areas could also be associated to a better diagnostic accuracy, as in 80% of our cases the final histological diagnosis was a grade III glioma.

Regarding brain metastasis, we could also confirm previous experiences (36), showing a very good tumor visualization in most of the cases with a percentage of GTR in 96% of the cases. Of notice, in 3 melanomas we did not find SF to be a useful adjunct, as the tumor was clearly visible under the microscope with white-light illumination.

We also found a good correspondence between pre-operative MRI enhancement and intraoperative fluorescence identification in in neuronal and mixed glioneuronal tumors, in other astrocytic tumors, in other types of neuroepithelial tumors and in lymphomas. SF was useful to identify pathological nodule, but also during surgery and at the end of resection, to verify the absence of residual tumor tissue. We administered fluorescein also in some cases of cranial and spinal schwannomas but, even if they showed an intense and homogeneous fluorescence, at the standard dosage fluorescein failed in distinguishing and identifying normal nerve fibers not related to the tumor. In addition, SF was considered not essential in hemangioblastomas that, despite the moderate but homogenous fluorescence both of nodular and fluid portions, present a peculiar and characteristic nodular component under white-light illumination, such to make not essential the use of fluorescein, at least with our protocol. On the contrary, we believe that a more appropriate application of fluorescein was proposed by Rey-Dios et al. (37) for these tumor subtypes. They reported the intravenous administration of fluorescein in bolus immediately before tumor resection to better visualize its vascular network, including arterial feeders, draining veins, and the nodule, as similarly reported by our group with ICG video-angiography (48, 49) (Table 1).

Two hundred and forty-five patients enrolled in the study were scheduled for surgery with a previous planning of macroscopic resection but keeping in mind the philosophy of maximal safe resection: in this series, we obtained a high percentage of GTR (181/245, 73.9%). Minimal residual tumor (lower than 10% of preoperative tumor volume) at postoperative MRI was expected in thirty-seven cases (15.1%), as it was involving eloquent areas or due to the adherences to major brain vessels or cranial nerves and was therefore independent from the use of fluorescein-guided technique. Conversely, in seventeen cases (6.9%), the residual tumor was an unexpected finding, based on the absence of clear residual intraoperative fluorescent tissue: this can be related, as for other fluorophores, to the fact that the residual tissue was not exposed during resection because hidden under the normal brain parenchyma. Finally, in the remaining ten cases (4.1%) the surgical resection was only partial due to the location in eloquent areas, or the significative encasement of major brain vessels or cranial nerves, especially for voluminous posterior fossa tumors (Table 1).

Twenty-eight patients were included in our protocol for fluorescein-guided biopsy. Regarding open biopsies, SF was used in four multifocal GBM, in five lymphomas and in five suspected vasculitis process/cerebral angiopathy with a cortico-subcortical component which was chosen as a target, in one pineocytoma with a voluminous cyst, after its surgical fenestration, and in one spinal PA, to identify the enhancing component for histopathological analysis. Regarding patients that underwent open biopsies, SF was always judged useful and, in every case, the tissue specimen was enough for an adequate histopathologic analysis. Regarding frameless/stereotactic biopsies, our series is limited to twelve patients, with a diagnostic accuracy of 75% (9/12): in particular, in two primitive multifocal gliomas, histopathological analysis revealed reactive gliosis despite the fluorescence of specimens, whereas in one recurrent thalamic glioma, the histology suggested choroidal plexus tissue, which can explain the SF enhancement of the samples, due to the absence of BBB, and physiological accumulation of SF in this cerebral area. In two of these patients, a fluorescein-guided diagnostic biopsy was repeated and was successful in obtaining a histological diagnosis (one AA and one oligodendroglioma). Although limited to 28 cases, these results seems to suggest, as already preliminarily shown in previous experiences (61), that SF could assist during biopsies of CNS lesions, for a correct identification of the best target for the histological diagnosis (Table 2).

Nobody, between patients of our series, presented fluorescein-related side effects or adverse reactions to SF administration, either singly or combined with other fluorophores. The only visible manifestation of intraoperative fluorescein administration is, as noted, the onset of transient yellowish stain of the urine, that rapidly disappear after 24–48 h : patients should be aware about this particular but totally harmless effect. We believe that the lack of any side effect, in particular any allergic reaction, was predominantly related to the low dosage used in this trial, thanks to the use of a dedicated filter into the microscope, that allowed a more accurate identification of fluorescent tissue, us suggested firstly by our group (27).

The main limitation of this study presented is represented by the lack of data about overall survival; in fact, the authors considered only a surrogate indicator which is the EOR, and by the lack of the direct comparison of surgery with and without SF aid due to ethical reasons. Furthermore, we did not make any comparison between the use of SF and other available fluorophores, like 5-aminolevulenic acid, that has been established as a surgical adjunct in surgery of HGG (20) and that has been reported as a possible advantage in selected cases of brain metastasis (62). Finally, the utility of SF in stereotactic/frameless biopsies is only descriptively considered without a proper statistical analysis, thus it is not able to establish a predictive value of SF. We have also to stress that, in most of the Countries, SF is still considered off label for neuro-oncological procedures. Thus, a widespread utilization of fluorescence-guided surgery will depend on the definitive approval by the competent authorities.

## Conclusion

On the basis of its unspecific mechanism of action, the use of SF as fluorescent tracer in neuro-oncology should be considered in several applications. Indeed, as these preliminary results seem to suggest, the same unspecific mechanism may also be seen as its strength since SF can be used as an operative adjunct in every CNS tumor with some degree of BBB disruption as detectable by neuroradiological imaging after contrast medium administration, but also by PET with amino acids positivity. Further studies are required to clarify the field of application of SF in neuro-oncological procedures and definitively assess the value of this technique on improvement of the EOR and overall survival.

## Ethics Statement

The FLUOCERTUM study has been approved by the Ethical Committee of the Fondazione IRCCS Istituto Neurologico Carlo Besta and written informed consent was obtained from all patients, in accordance with the Declaration of Helsinki.

## Author Contributions

JF, CC, and FA: study concept and design. JF, CC, IV, and FA: critical revision of the manuscript for intellectual content. All authors: acquisition of data, data analysis, and interpretation. PF and FA: study supervision.

### Conflict of Interest Statement

FA received honoraria from the Carl Zeiss Meditec Company for lectures at International Congresses. The remaining authors declare that the research was conducted in the absence of any commercial or financial relationship that could be constructed as a potential conflict of interest.

## Abbreviations

AA: anaplastic astrocytoma
AO: anaplastic oligodendroglioma
AIFA: agenzia italiana del farmaco (Italian Medicine Agency)
BBB: blood brain barrier
c.e.: contrast enhancement
CNS: central nervous system
CT: computerized tomography
EOR: extent of resection
FET: fluoro-ethyl-tyrosine
GBM: glioblastoma
GTR: gross total resection
HGG: high-grade glioma
ICG: indocyanine green
i.v.: intravenous
LGG: low-grade glioma
MET: 11C-methionine
MRI: magnetic resonance imaging
PA: pilocytic astrocytoma
PET: positron emission tomography
PR: partial resection
SF: sodium fluorescein
STR: subtotal resection
5-ALA: 5-aminolevulenic acid.
